# A Simple State-Determined Model Reproduces Entrainment and Phase-Locking of Human Walking

**DOI:** 10.1371/journal.pone.0047963

**Published:** 2012-11-12

**Authors:** Jooeun Ahn, Neville Hogan

**Affiliations:** 1 Department of Mechanical Engineering, Massachusetts Institute of Technology, Cambridge, Massachusetts, United States of America; 2 Department of Brain and Cognitive Sciences, Massachusetts Institute of Technology, Cambridge, Massachusetts, United States of America; McMaster University, Canada

## Abstract

Theoretical studies and robotic experiments have shown that asymptotically stable periodic walking may emerge from nonlinear limit-cycle oscillators in the neuro-mechanical periphery. We recently reported entrainment of human gait to periodic mechanical perturbations with two essential features: 1) entrainment occurred only when the perturbation period was close to the original (preferred) walking period, and 2) entrainment was always accompanied by phase locking so that the perturbation occurred at the end of the double-stance phase. In this study, we show that a highly-simplified state-determined walking model can reproduce several salient nonlinear limit-cycle behaviors of human walking: 1) periodic gait that is 2) asymptotically stable; 3) entrainment to periodic mechanical perturbations only when the perturbation period is close to the model's unperturbed period; and 4) phase-locking to locate the perturbation at the end of double stance. Importantly, this model requires neither supra-spinal control nor an intrinsic self-sustaining neural oscillator such as a rhythmic central pattern generator. Our results suggest that several prominent limit-cycle features of human walking may stem from simple afferent feedback processes without significant involvement of supra-spinal control or a self-sustaining oscillatory neural network.

## Introduction

Understanding the essential processes underlying human locomotion remains a central problem of motor control neuroscience and biomimetic robotics. The importance of this question goes beyond its scientific interest: a more profound understanding of human locomotor control will facilitate refinement and optimization of exoskeletal assistive devices to augment human walking or robotic therapy to aid locomotor recovery after injury.

One puzzling aspect of human locomotor control is the co-existing evidence pointing to different control architectures. Walking in unimpaired adults exhibits a repeatable spatial trajectory of the foot [1]. Presented with surface irregularity, subjects adjusted their minimum toe clearance by subtle modification of lower-limb kinematics [2]. Patients with spinal cord injury (SCI) who recovered following body-weight-supported treadmill training showed a foot trajectory that was close to the normal pattern, although they used obviously different joint coordination patterns [3]. These studies suggest that supra-spinal processes are predominant, adjusting peripheral muscle activation and joint recruitment to control the kinematics of the foot.

In contrast, robotic experiments and theoretical studies have provided compelling evidence that nonlinear limit-cycle oscillators without a vestige of central kinematic planning or control are competent to exhibit stable bipedal walking. With no sensing, actuation or control, the so-called passive dynamic walkers can provide a startlingly humanlike mimicry of bipedal walking; interaction between the inertial and gravitational mechanics of their limbs and intermittent impacts with the ground produce remarkably coordinated walking on a gentle slope [4], [5]. Of course, biological locomotion also involves neural processes but not necessarily kinematic planning and control. Unequivocal evidence of a rhythmic central pattern generator (CPG) underlying locomotion has been found in various vertebrates [6], [7], [8], [9], [10]. Stable rhythm generation requires a nonlinear limit-cycle oscillator and theoretical studies have demonstrated that CPG-driven bipedal walking is stable and hence a plausible mechanism of human locomotion [11], [12].

However, the contribution of a CPG to human locomotion is still unclear. Human infants show a primitive rhythmic stepping reflex, but the reflex typically disappears at about 6 weeks after birth [13]. When toddlers acquire independent walking at about a year old, they are not initially able to generate the rhythmic pattern of mature walking and this cannot be ascribed to immature postural control [14]. Rhythmic activation of peripheral musculature was evoked by non-rhythmic electrical stimulation of the lumbar spinal cord in patients with chronic spinal cord injury, but the relevance of that study to unimpaired human locomotion is unclear because altered descending neural excitation of the spinal cord almost certainly changes its excitability and may exaggerate the role of spinal circuits [15]. For unimpaired humans, continuous (non-rhythmic) leg muscle vibration produced locomotor-like stepping movements, and non-rhythmic spinal electromagnetic stimulation applied to unimpaired human vertebrae induced involuntary locomotor-like movements [16], [17]. However, subjects in those studies were suspended in a gravity-neutral position, unlike normal walking, making it difficult to generalize the results to upright walking. To the best of our knowledge, it is entirely possible that any evidence of a CPG underlying human walking may be residual, a legacy of phylogenetically earlier mechanisms of locomotion, since superseded, that are unimportant in the control of locomotion in modern humans.

A further source of confusion is the variety of mechanisms that may generate limit-cycle behavior in human walking. The success of passive dynamic walkers shows that mechanical interaction between the periphery and the environment is sufficient to demonstrate stable periodic gaits on a slope, but active control is necessary to yield periodic gaits on level ground or up a slope. Input from a rhythmic pattern generator may enable stable bipedal walking, but stable periodic gaits on level ground are also achievable with minimal feedback control by simple state-determined actuation as in the Cornell biped [11], [12], [18], [19]. Even for vertebrates with clear evidence of spinal pattern generators, afferent sensory input is critical for locomotion [20], [21]. Generating the human locomotor pattern depends on load-related input, hip afferent input and location-specific information from the skin of the foot [22], [23].

Recently we reported behavioral evidence that some form of nonlinear limit-cycle oscillator plays a measurable role in unimpaired human walking [24]; we applied periodic torque pulses to the ankle of walking subjects at periods different from their preferred cadence, and the gait period of 18 out of 19 subjects entrained to this mechanical perturbation, converging to match that of the perturbation. Significantly, entrainment occurred only if the perturbation period was close to subjects' preferred walking cadence: it exhibited a *narrow basin of entrainment*. Further, regardless of the phase within the walking cycle at which perturbation was initiated, subjects' gait synchronized or *phase-locked* with the mechanical perturbation at the end of double stance where ankle actuation occurs.

In the study reported here we develop a minimal mathematical model that is competent to quantify the observed limit-cycle behaviors of unimpaired human locomotion—stable periodic motion, entrainment to periodic perturbations, and phase-locking. Because the kinematics and dynamics of the human neuro-mechanical system are inordinately complex, our goal was to avoid clutter and its attendant confusion and focus only on essential features that might give rise to observed behavior. To maximize simplicity the entire human musculo-skeletal system was modeled as a point mass with massless legs as in the spring loaded inverted pendulum (SLIP) models [25], [26]. However, our model is fundamentally different from energy conservative SLIP models; in our model, foot-ground interaction dissipates the energy of the system as in human walking, which leads to critical differences in the model's competence. Our model is also distinct from the classical compass gait bipeds [27], [28], [29] in that our model has a double stance phase as in human walking, whereas the compass-gait bipeds do not. In addition, analytical solution is intractable for the compass-gait biped due to its complexity, whereas the simplicity of our model enabled analytic expressions for several key model behaviors. In the following we show that a simple model with 1) one degree of freedom, 2) without supra-spinal control and 3) without a self-sustaining oscillator like a spinal pattern generator can successfully reproduce observed behaviors. This suggests that a simple state-dependent controller using afferent feedback may serve as a minimal component model of human walking dynamics.

## Model

### General Description

**Figure 1:**
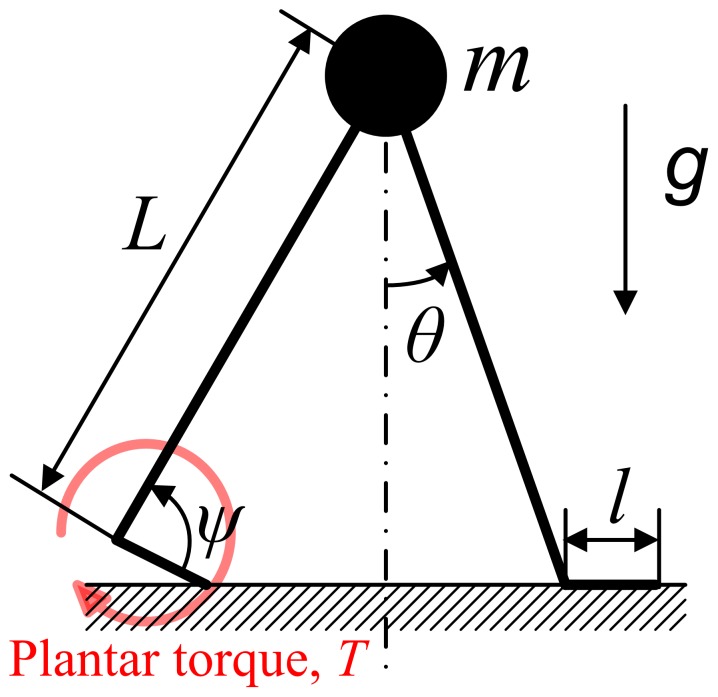
A schematic of the walking model. A point mass is restrained by rigid massless legs. The trailing ankle is actuated as a cocked (pre-loaded) spring released at the beginning of double stance. The hip joint and the leading ankle do not exert any torque.

A schematic of the model defining its variables and parameters is shown in Fig. 1. A point mass moves in a vertical plane under the influence of gravity, restrained by rigid massless legs. The swing leg can be moved instantaneously in front of the mass. Scuffing (contact of the swing leg with the ground) is ignored. Each leg has two joints—a hip and an ankle. Ankle actuation provides propulsion whereas the hip joint is assumed to be a frictionless pivot, which cannot apply any torque. However, we assume that the angle between the legs is always reset as 2*α* at the beginning of a step. Due to the assumption of massless legs, resetting the angle between the legs does not consume any energy.

**Figure 2:**
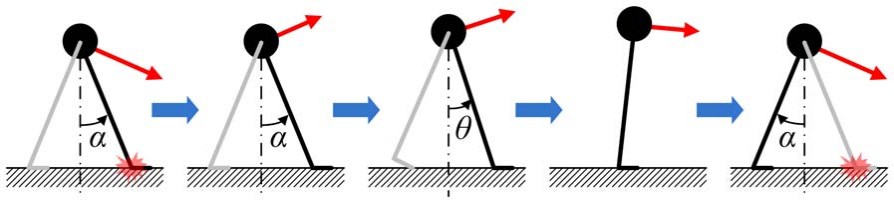
One step cycle of the walking model. The end and beginning of a step is the moment when the leading foot collides with ground. During double stance the model moves as four linked bars. During single stance the model moves as an inverted pendulum.

Sequential configurations of the model during one step cycle are depicted in Fig. 2. At the collision of the leading foot with the ground, the velocity of the point mass changes instantaneously. Immediately after the collision, the model is in double stance and the trailing leg ankle is actuated. During double stance the model behaves as an actuated four-bar linkage. The ankle of the leading leg acts as a hinged joint during double stance and the following single stance phase. We assume that trailing-leg ankle torque during double stance is determined by a linear torsional spring as
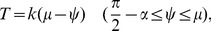
(1)where *T* is plantar ankle torque at the trailing ankle, *k* is stiffness, a constant, *ψ* is ankle angle that is positive towards plantar flexion as depicted in Fig. 1, and *μ* is maximal plantar flexion angle. The torque becomes zero when *ψ* reaches *μ*. By virtue of the zero mass of the feet, the trailing foot pushes on the ground only as long as the actuation torque is positive; double stance ends at the moment when the ankle torque becomes zero, or equivalently when *ψ* reaches *μ*. During the following single stance, there is no actuation torque, and the dynamics of the swing leg is irrelevant because it has no mass; the model acts like an inverted pendulum hinged at the ankle of the stance leg. A step cycle ends when the hip angle *θ* reaches −*α*, its value at the foot-ground collision.

### The Equations of Motion

**Figure 3:**
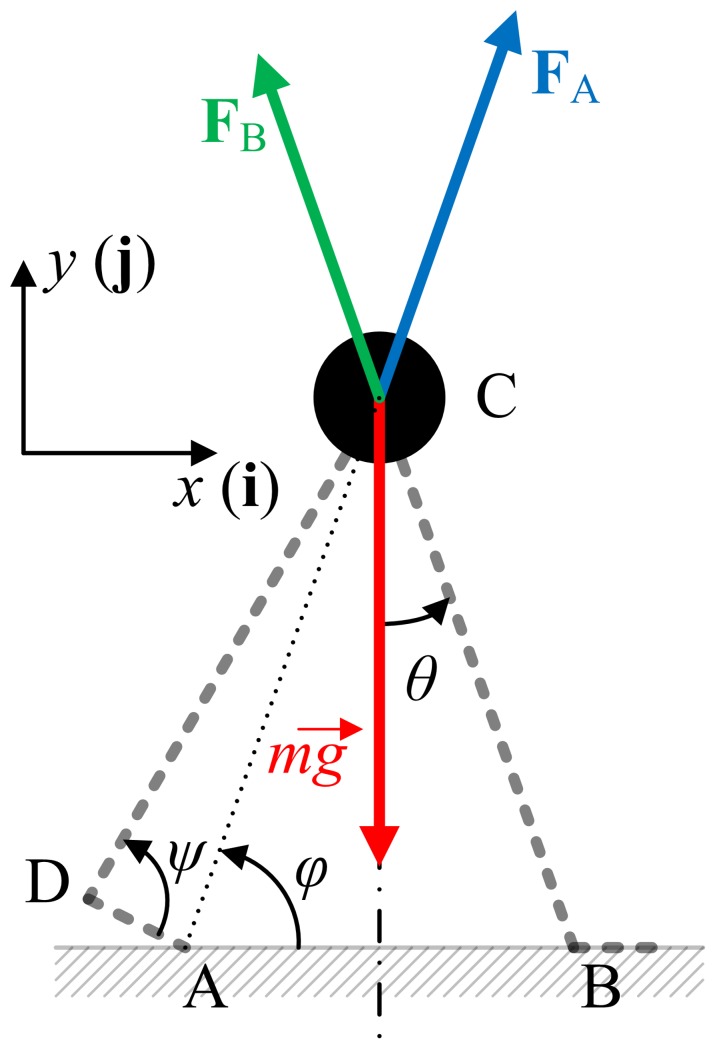
A free body diagram of the model during double stance. Points A, B, C, and D denote the toe of the trailing leg, the heel of the leading leg, the point mass, and the trailing ankle respectively. The angle between the horizontal line and the line AC is defined as *φ*.

A free body diagram during double stance is shown in Fig. 3. To simplify the analysis, the ground reaction forces are assumed to be concentrated on the trailing toe (point A) and the leading heel (point B). Due to zero mass of the legs, the ground reaction force applied at each leg directly points the point mass (point C). The ground reaction forces at A and B are denoted as **F**_A_ and **F**_B_, respectively. The angle between the horizontal line and the line AC is defined as *φ*.

Describing the motion of the point mass with respect to the leading heel, B,
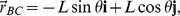


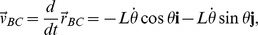
and
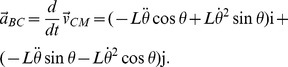
Using the linear momentum principle in both horizontal and vertical directions,
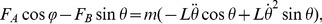
(2)and
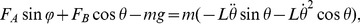
(3)yielding
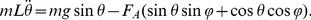
(4)Considering zero mass of the trailing foot (segment AD in Fig. 3), plantar ankle torque should be balanced with the torque due to the ground reaction force, **F**_A_.
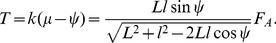
(5)From Eq. 4 and Eq 5, the equation of motion becomes
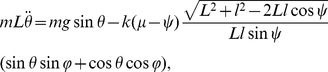
(6)where the angles, *ψ* and *φ* are functions of *θ* as
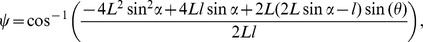
and
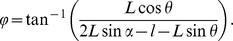

Eq 6 is the equation of motion of the model during double stance. During single stance, the equation of motion is the same as that of an inverted pendulum, which is
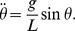



### Ground Reaction Forces

To simulate physically feasible walking, it is important to evaluate the ground reaction forces and investigate whether they remain positive.

#### Ground Reaction Forces during Double Stance

As shown in Eq 3, *F*_A_ is positive as long as the ankle torque is applied the in plantar direction. Therefore, *F*_A_ is positive throughout double stance. However, *F*_B_ can be negative if 1) *F*_A_ is so excessive that the mass *m* is lifted regardless of gravity, or 2) the velocity is so excessive that the mass *m* is lifted due to centrifugal force. Therefore, it is necessary to investigate the explicit form of *F*_B_. Subtracting Eq 3 multiplied by cos*θ* from Eq 2 multiplied by sin*θ*,
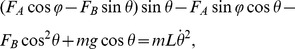
or
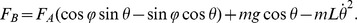
Using Eq 5, *F*_A_ can be expressed as a function of *ψ*, yielding
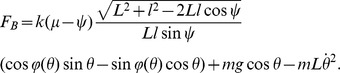
The time course of this reaction force can be numerically evaluated.

#### Ground Reaction Forces during Single Stance

The single stance phase is simply a motion of an inverted pendulum, and the condition that keeps the model from flying off is to keep 
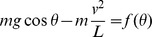
 above zero. The function *f*(*θ*), which equals to the magnitude of the ground reaction force, has the minimum at the end of the single stance phase because cos*θ* has the minimum at *θ* = −*α* with the given range of motion, and *v* ^2^ has the maximum at the same moment of *θ* = −*α* during the single stance phase. Therefore, the sign of *f*(−*α*) concludes whether the model flies off or not.

Rewriting the condition, to prevent the model from flying off the ground,
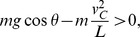
where *v_C_* is the speed of the point mass just before a collision.

### Parameter Values

Parameter values are summarized in Table 1. Leg length, foot length, maximal plantar extension angle, hip angle at foot-ground contact and mass were chosen to approximate morphological data of human adults. The value of ankle actuation stiffness was chosen to match the maximal ankle torque of the model with that of normal human walking. Experimental data shows that peak plantar-flexion torque in normal gait is approximately 17% of body weight×leg length [30]. For the model, this value corresponds to 133.4 (N-m) and to match peak ankle torque at the beginning of a double stance with this value, *k* was determined to be 87.3 (N-m/rad).

**Table 1:** Parameter values for the ankle actuated model.

Parameter	Meaning	Value
*m*	mass	80 kg
*L*	leg length	1 m
*l*	foot length	0.2 m
*g*	gravitational acceleration	9.81 m/s^2^
*α*	angle of the leg at heel strike	π/6 rad
*μ*	maximal plantar extension of the ankle	2.576 rad
*k*	ankle actuation constant	87.3 N·m/rad

### Analysis Method

In this study, we investigated whether the model was able to reproduce salient features observed in normal human walking: 1) existence of a period-one gait; 2) stability of this period-one gait; 3) entrainment of this period-one gait to periodic mechanical perturbations with a finite basin of entrainment; and 4) phase locking so that the perturbation occurred at the end of double stance. Because of the extreme simplicity of the model, most of these questions could be addressed by a straightforward application of calculus and algebra. Additional results were obtained by numerical simulation implemented in Matlab using the Simulink toolbox (Mathworks Inc.). Numerical integration by the Runge-Kutta method was performed with a fixed step size of 10^−4^ and absolute and relative error tolerances of 10^−6^. The validity of the numerical simulation was checked using either available analytical solutions or by repeating simulations with a tenfold smaller tolerance. The method used was precise enough to deal with the discontinuities in the model; the *Floquet multiplier* (explained in Results) evaluated from numerical simulation was 0.25000 whereas the analytical solution yields cos^2^2α, which is 0.25 with the given parameters.

## Results

### Step-to-Step Function

To analyze existence and stability of a periodic gait, we used the concept of a step-to-step function used by Bauby and Kuo [31] whose input and output are state variables at the beginning of one step and at the beginning of the next step respectively. In the language of dynamical systems, a step-to-step function is a discrete Poincaré map, and a period-one gait is a fixed point of the Poincaré map. With the simplification of perfect symmetry, we allowed one step to represent one cycle. In real bipedal locomotion which may include asymmetry, one period of human locomotion corresponds to one stride, which consists of two steps. As the model has only one degree of freedom (*θ*), and the dynamics of the model can be fully described with a 2^nd^ order ordinary differential equation, evolution of the system can be described in two dimensional state space 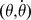. If the beginning of one cycle is defined as the moment of a foot-ground collision, or equivalently as the moment when *θ* reaches −*α*, the step-to-step function is defined as 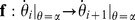. The existence of a period-one gait requires that 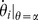 satisfies 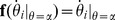, and the local asymptotic stability of this period-one gait is established if the derivative of the step-to-step function evaluated at the period-one gait satisfies 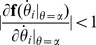. Note that for this model, because the step-to-step function is defined in one dimensional space, the derivative of the step-to-step function is not a matrix but a scalar, which is equivalent to a *Floquet multiplier*.

### Existence of a Period-one Gait

The actuation torque, *T* is a function of *ψ*, which is determined by *θ* and the geometry of the model. Consequently, the work done by the ankle torque can be written as 
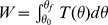
, where *θ_0_* and *θ_f_* indicate the value of *θ* at the beginning and the end of a double stance respectively. The work done per step is a constant because *θ_0_* and *θ_f_* in each step are constants. Equivalently, the potential energy initially stored in the ankle spring, which is released during double stance, is determined by the hip angle *α*, the spring stiffness *k*, and the maximal plantar flexion *μ*, all constants. On the other hand, a foot-ground collision reduces the speed of the model by a factor of cos2*α*, and therefore reduces kinetic energy by cos^2^2*α*. (At collision, this model is a special case of the “rimless wheel” models; a detailed explanation of this speed reduction due to collision is presented in [32].) Taken together, for the model to exhibit a period-one gait, the loss of kinetic energy due to a foot-ground collision must be exactly compensated for by the work done by the ankle torque. For this, the speed of the point mass just before a collision, *v_C_*, and the corresponding 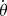, denoted 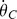, must satisfy
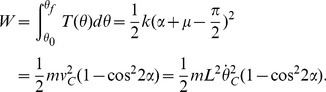
(7)This expression has two solutions differing only in sign. The negative solution corresponds to forward progression; 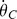 for a period-one gait is
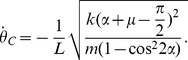
(8)For a period-one gait to exist, two additional conditions must be satisfied: 1) the point mass must have enough kinetic energy at the beginning of single stance to “vault over” to make the next step, and 2) the ground reaction forces must not be negative, i.e. the model must not “fly off” the ground. These two conditions limit the range of *k*. Excessively small *k* cannot supply enough energy to make the model vault over. Conversely, with overly large *k*, the leading foot is lifted during double stance by an excessive ground reaction force at the trailing foot. A closed-form expression for the lower limit of *k* (the “just-vault-over” stiffness) can be obtained analytically. Let the marginal stiffness be *k*_C_. In the case in which the model just vaults over, the kinetic energy of the model becomes zero at the apex of θ = 0. Using the work-energy principle,
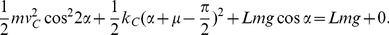
(9)From Eq 7 and Eq 9,
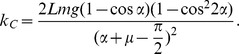
With the parameter values given in Table 1, *k_C_* became 67.5 (N-m/rad); *k* was greater than *k_C_*, which satisfied condition 1). To check that *k* was less than its upper limit (the “just-fly-off” stiffness), we evaluated the ground reaction forces numerically with the selected parameter values. The minimum ground reaction force at the leading heel occurred at the beginning of double stance, and was evaluated as 149.5 N; the ground reaction forces did not go below zero, satisfying condition 2). Thus the existence and uniqueness of a period-one gait were established. The step period of this gait, *τ*_0_, was 0.967 (s) and the average forward speed was 1.03 (m/s), comparable to freely-selected low-speed human walking (0.694 (s) step period and 0.92 m/s on average) [33].

### Asymptotic Stability of the Periodic Gait

**Figure 4:**
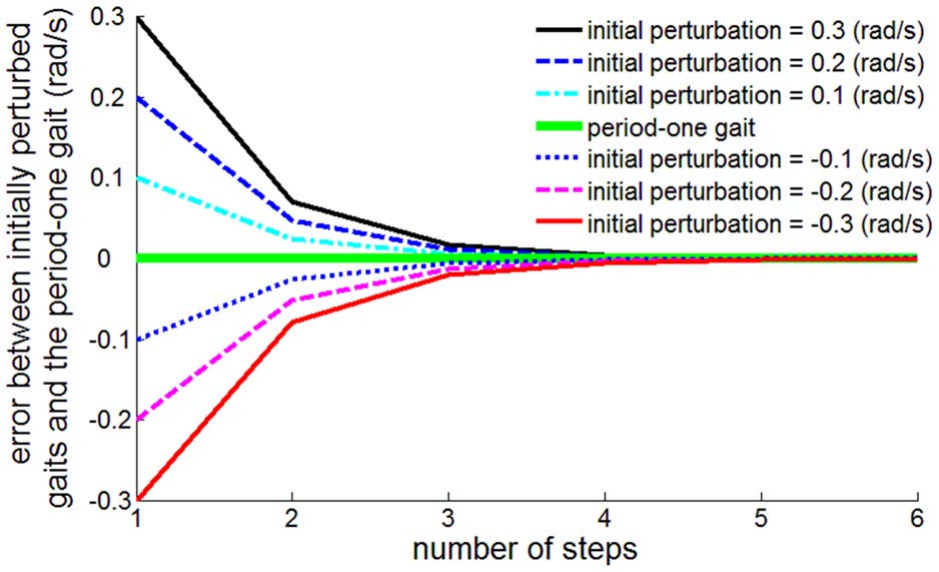
Asymptotic stability of period-one gait. Errors in initial conditions of angular velocity converge to zero as the number of steps increases.

Asymptotic stability of the period-one gait can be established analytically. Let a collision occur at *t* = 0, and the next collision occur at *t* = *t_f_*. Also, let *t* = *t_f_*_+_, *t* = *t_f_*_−_ and *t* = 0_+_ indicate the moments right after *t* = *t_f_*, just before *t* = *t_f_* and right after *t* = 0, respectively. Using the work-energy principle, 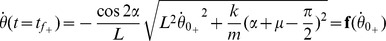, where 
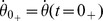
. The derivative of the step-to-step function **f** becomes
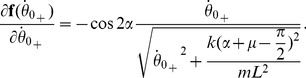
(10)From Eq 8, 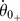 at the fixed point (the period-one gait) becomes
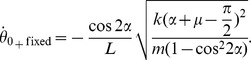
Substituting into Eq 10, the derivative of the step-to-step function at the fixed point is cos^2^2*α*. With the parameter value of α = π/6 (rad), this becomes 0.25, substantially less than unity, which guarantees local asymptotic stability of the fixed point of the step-to-step function. Numerical evaluation yielded the same value, validating the numerical methods. Simulations demonstrating the asymptotic stability of the period-one gait with the selected parameter values are shown in Fig. 4.

### Entrainment of the Period-one Gait

To investigate the competence of the model to reproduce entrainment to mechanical perturbations, we superimposed periodic plantar-flexion torque pulses on the ankle (in addition to the torque due to the ankle actuation). In the previous experimental study [24], periodic square torque pulses of magnitude 10 N-m and duration 0.1 second were applied to one ankle of a walking subject at periods that differed slightly from the subject's preferred stride period. A magnitude of 10 N-m is approximately comparable to 10% of maximum ankle torque during normal walking in male adults. For comparison with the experimental study in which a perturbation pulse was applied to one ankle, in the model the period of the perturbation torque, *τ*_p_ was set to be close to the stride period of the model's unperturbed gait, 2*τ*_0_; the range of *τ*_p_ was 2*τ*_0_±0.1 second, which covered a range of perturbation periods similar to the experimental protocol in [24]. The amplitude of the added torque pulse was 10% of the maximal ankle torque of the model, and the pulse width was 0.1 second as in the experiment. By virtue of zero mass of the legs, a perturbation cannot contribute to the dynamics of the model when the perturbation torque is applied to an ankle in swing phase. Any portion of the perturbation pulse that was applied to an ankle in swing phase was nullified. As a result, the torque to the trailing ankle under periodic mechanical perturbations became
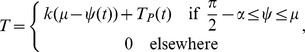
where
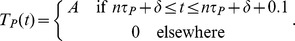
The constant *A* is the amplitude of the perturbation pulse, *n* is an arbitrary positive integer or zero, τ_p_ is a perturbation period, and *δ* is the initial phase of the perturbation, which can be an arbitrary constant.

**Figure 5:**
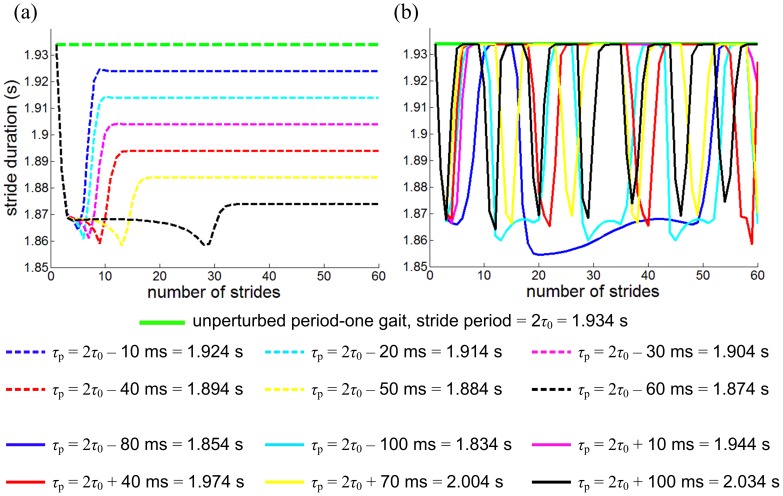
Entrainment to mechanical perturbations with a finite basin. Stride period is plotted as a function of stride number; (a) shows entrained gaits, and (b) shows gaits that failed to entrain. For entrained gaits, the stride period converged to the perturbation period, *τ*_p_, whereas stride period continued to fluctuate when gait was not entrained. Note that the model shows a narrow basin of entrainment. Any perturbation with *τ*_p_>2*τ*_0_ or *τ*_p_≤2*τ*_0_−80 (ms) did not entrain the model.

Entrainment of the model to the perturbations was observed with a narrow basin of entrainment (Fig. 5); the model entrained to *τ*_p_ only if *τ*_p_ was close to 2*τ*_0_. The basin of entrainment was approximately 3.93% of 2*τ*_0_, which is within one standard deviation of the estimated basin of entrainment observed in the experiment (6.7%±3.6% of unperturbed stride period) [24].

### Phase Locking at the End of a Double Stance

**Figure 6:**
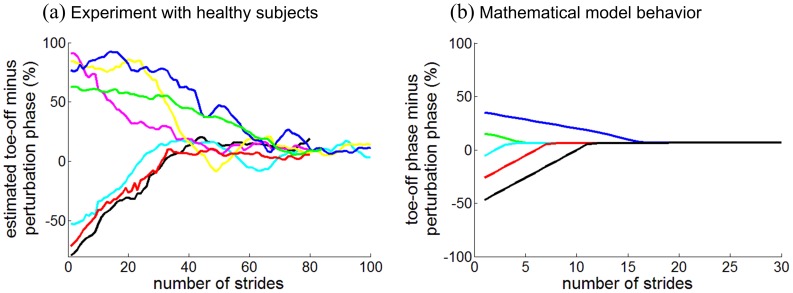
Phase-locking at terminal stance of normal human walking and the mathematical model. In (a), the experimental data of normal subjects from [24] are shown. The estimated phase difference between toe-off (initiation of swing) and the initiation of the perturbation pulse is plotted as a function of stride number. In (b), the phase difference between toe-off of the model and the initiation of the perturbation pulse is plotted for entrained gaits with (*τ*_p_ = 2*τ*_0_−50 ms) and various initial phases of the perturbation pulse. In both (a) and (b), regardless of the initial phase, the perturbation pulse converged to a phase close to toe-off; the model successfully reproduced the phase-locking at the end of double stance which was observed in the experiment.

**Figure 7:**
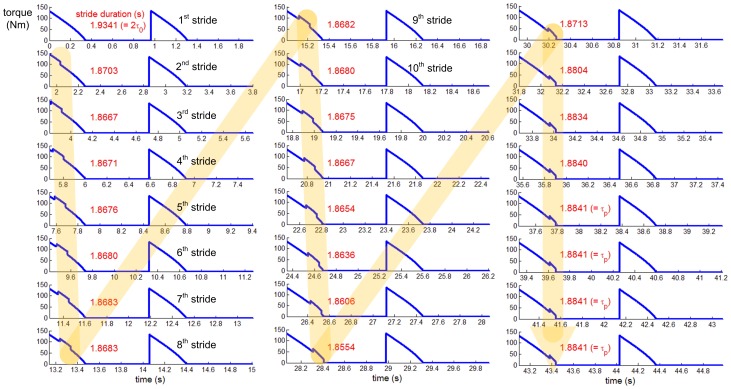
Torque profiles at the trailing ankle during successive cycles. Time profile of torque to the ankle is plotted per stride when a perturbation with period of 1.8841 (s) (τ_p_ = 2τ_0_−50 ms) is applied. The perturbation pulses, which are superimposed on the intrinsic ankle actuation, drift along the gait cycle, but eventually phase lock at the end of double stance where the intrinsic ankle actuation torque approaches zero. The evolution of stride durations (displayed in red numbers) shows that the stride duration converges to the perturbation period; entrainment is achieved.

In addition to reproducing entrainment with a finite basin, the model also reproduced the phase locking that we observed in unimpaired human walking [24]. In particular, the perturbation pulse converged to the end of double stance regardless of the gait phase at which the perturbation pulse was initiated (Fig. 6). Furthermore, as shown in Fig. 6, the time-course of phase-locking bore a clear qualitative resemblance to the pattern observed in the experiments [24]. The torque profiles at the trailing ankle during successive cycles are shown in Fig. 7. The perturbation pulse was clearly phase locked at the end of double stance.

## Discussion

Despite its intentionally extreme simplicity, the model presented here reproduced all of the following features observed in normal human walking: 1) a periodic bipedal walking pattern; 2) local asymptotic stability of that periodic walking pattern; 3) entrainment of that walking pattern to periodic mechanical perturbations with a narrow basin of entrainment; and 4) phase locking to locate the perturbation at the end of double stance when entrained. The extreme simplicity of the model must be emphasized. All of the complex biomechanics of the human musculo-skeletal system (on the order of 600 muscles activating about 200 degrees of freedom) was distilled into a model with only one degree of freedom. Though those additional complexities no doubt contribute to unimpaired locomotion, our results show the competence of this simple model to reproduce observable features of sagittal-plane human locomotor dynamics.

An important detail of this model is that it involves afferent feedback: actuation of the trailing-leg ankle is triggered based on the system state, and the control of the angle of leading leg before foot ground collision requires feedback. The afferent information required for the triggered actuation might be derived from foot contact of the leading leg, reflecting cutaneous input or load-related afferents, e.g. from Golgi tendon organs. Alternatively, it might be derived from stretch receptors, for example those that signal hip extension; or it may arise from a combination of these sources. The information required for control of leading leg angle might also be obtained from proprioceptive sensory feedback. However, the model deliberately omitted any self-sustaining intrinsic neural oscillator such as a CPG. It also omitted supra-spinal control specifying limb kinematics. Though either or both of these factors plausibly contribute to unimpaired human locomotion, the competence of our model suggests that they may be non-essential to reproduce observed features of normal human walking—stable periodic oscillation, entrainment and phase-locking—that may instead emerge from the nonlinear dynamics of the neuro-mechanical periphery.

### Energy Dissipation Plays a Key Role in Asymptotic Stability

The simplicity of our model facilitates physical interpretation of the results: though afferent feedback is included, simple mechanics account for much of the model behavior. The existence and stability of the model's period-one gait can be explained physically. The model was designed to be energetically identical to a rimless spoked wheel on a slope, perhaps the simplest passive dynamic walker [32]. The amount of work done by ankle torque is constant per step, but the loss of kinetic energy due to foot-ground collision is proportional to the square of speed. Faster collisions dissipate more energy and the model slows down; slower collisions dissipate less and the model speeds up. Similarly, for a passive dynamic walker on a slope, gravity supplies a fixed amount of energy per step, but foot-ground collision dissipates kinetic energy in proportion to square of speed. A constant energy added per step combined with energy loss per step proportional to square of speed yields asymptotically stable periodic motion.

It is important to note that the dissipation of kinetic energy due to foot-ground collision is the essential source of locally stable entrainment (i.e. with a narrow basin of attraction) of this model. It also gives rise to the asymptotic stability of the model's unperturbed periodic gait. Many widely-cited (indeed “classical”) studies have assumed that animal locomotion evolved to consume the least energy [34], [35], [36], [37], [38], [39]. Though the value of minimizing the energy cost of transportation is self-evident, robust stability arguably takes a higher priority. The derivative of the step-to-step function (cos^2^2*α* in our model) determines the strength of the asymptotic stability. It is also directly related to the reduction of kinetic energy due to foot-ground collision. Provided the parameter values admit a periodic gait, greater energy dissipation per step yields stronger stability and vice versa. The extreme example of high energy efficiency but marginal stability is pure rolling on level ground. In our model, as *α* approaches zero, step size becomes infinitesimal and the behavior approaches pure rolling. However, although the energy cost of transportation approaches zero in this limit, stability also becomes marginal as the *Floquet multiplier* cos^2^2*α* approaches unity.

Despite its simplicity, our model may represent fundamental aspects of the peripheral neuro-mechanics of legged animals. To the extent that it does, it demonstrates a trade-off between energy efficiency and stability that appears to be a fundamental feature of legged locomotion. Evolution may be regarded as optimizing the probability of reproduction, supported by optimizing survival (at least until reproduction) [40]. Reliable performance (e.g. robust locomotor stability) may therefore take higher priority than energy efficiency. In our model, stability requires energy dissipation; strictly minimal energy consumption implies marginal stability. At a minimum stability (which requires energy dissipation) should be included in the function to be optimized. Although collision-free legged locomotion is physically possible, to the best of our knowledge non-elastic interaction between foot and ground, which dissipates kinetic energy, is a common characteristic of legged animal locomotion. In human locomotion muscles do more positive than negative work even when walking at constant average speed on level ground [41]. This provides evidence of energy dissipation (e.g., due to the non-elastic interaction between a foot and ground) in normal human walking.

### Limitations of Conservative Walking Models

Energy conservative walking models are appealing; they serve as a “gold standard” for minimal energy consumption in legged locomotion, and have nice mathematical properties. Unfortunately, they *cannot* reproduce the entrainment to periodic perturbation that was observed. Nor can they reproduce the robust stability of animal locomotion. Some studies have reported stable limit-cycles in energy-conservative models [26], [42], [43]. However, their stability can neither be robust nor *asymptotic* in response to an arbitrary perturbation, however small. By definition, a conservative model has no means to recover from a change of its energy level. If the model suffers a perturbation that changes its energy level, its limit-cycle is marginally stable at best. Of course, the probability that an arbitrary perturbation will not change system energy is zero; practically every perturbation changes the energy level. No conservative model is able to exhibit the asymptotic stability which appears to be one of the most fundamental properties of animal locomotion. Most important for the purpose of this paper, no conservative model can reproduce the entrainment to periodic mechanical perturbations with phase-locking that we observed in unimpaired human walking [24]. In a conservative model, a perturbation such as we applied that occurred late in the double stance phase would necessarily increase the system energy. With phase locking such as we observed, system energy would increase on every stride and grow without bound. That is clearly incompatible with our experimental observations.

### Finite Work Done by a Perturbation Determines a Finite Basin of Entrainment

**Figure 8:**
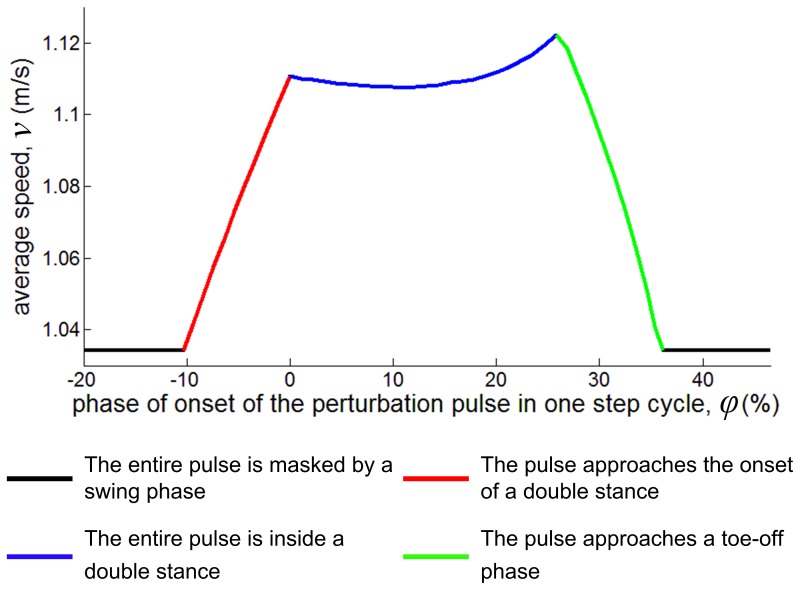
The average speed of the model (step length/step duration), *v*, vs. the phase of a perturbation pulse, *φ* for a pulse of constant amplitude and duration. When a pulse is located in a swing phase, it cannot accelerate the model, and *v* is lower than when the entire pulse is inside double stance. Average speed, *v*, increases as *φ* approaches 0, the onset of double stance, and decreases as *φ* approaches the end of double stance.

The average speed of the model (step length/step period), *v*, is plotted as a function of the perturbation pulse initiation phase, *φ*, in Fig. 8. The minimum average speed is that of period-one gait without a perturbation. It occurs when all of the perturbation pulse is applied within the swing phase. The model cannot walk more slowly because the mechanical perturbation, which applies to the trailing ankle, only accelerates the system. The maximum average speed occurs when all of the perturbation pulse is applied within the double-stance phase. It is upper-bounded because the amount of the acceleration due to the perturbation is limited. The range of average speeds under perturbation is less than 9% of the minimum (unperturbed) average speed. The limited amount of energy supplied by the mechanical perturbation results in the small range of average speeds, and therefore, with a fixed step length determined by *α*, determines a finite basin of entrainment to periodic perturbations.

### Phase Locking Occurs at the End of Double Stance

Entrainment only requires the walkers' cadence to converge to the perturbation period; the perturbation pulse will then occur at a constant phase in the entrained gait, but it may be at any constant phase. Remarkably, in experiments with unimpaired humans, we observed phase-locking for all entrained gaits such that the perturbation occurred at the end of double stance [24]. The model presented here reproduced that observation which may be understood using the *v* vs. *φ* curve of Fig. 8. If a perturbation pulse is entirely contained in a swing phase, it cannot accelerate the model. The average speed increases as the perturbation phase approaches 0, the onset of double stance, because a progressively larger portion of the pulse occurs in the double stance phase, where it can accelerate the model (the red line in Fig. 8). Conversely, the average speed decreases when the pulse approaches the end of the double stance phase because a progressively larger portion of the pulse occurs during the following swing phase, where it can do no work (the green line in Fig. 8). Consequently, the *v* vs. *φ* curve has a positive slope at the onset of a double stance, and a negative slope at the end of a double stance.

If the model is entrained to a periodic perturbation its cadence is same as the perturbation period. Any small variation that accelerated the model would make the perturbation pulse occur at a later phase in the following stride. In other words, *φ* increases on the next stride if the model accelerates. Near the end of double stance, if the model accelerates, increased *φ* decreases speed on the next stride because 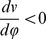. A similar argument applies if any small variation decelerated the model; the negative slope of *v* vs. *φ* stabilizes the entrained gait.

The *v* vs. *φ* curve (Fig. 8) also has a negative slope in the earlier portion of double stance. However, the negative slope of this region may be ignored for the following reasons. First, the negative slope in this region is much smaller than at the end of double stance; the strength of the stability is much weaker. Second, this region allows only an extremely narrow basin of entrainment, less than 0.13% of 2*τ*_0_. This region could account for only a limited fraction of the entrained gaits which occupied a basin more than 30 times wider, both in the model (Fig. 5) and the experiment [24]. Finally, the negative slope within the double-stance phase is sensitive to the details of the model, especially the ankle torque profile (Eq. 1). With a different ankle actuation profile, the portion of a double stance with a negative slope may move, shrink, or even vanish. In contrast, the negative slope at the end of double stance is always evident as long as we make the physically reasonable assumption that a torque pulse at the ankle provides no propulsion when the leg is in swing phase.

The mechanism of stability described above also makes the end of double stance an *attractor* for phase locking. If the model speed is lower than that of an entrained gait, *v*_entrained_, the next perturbation pulse will occur at an earlier phase, and the speed will increase toward *v*_entrained_ due to the negative 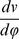. If the model speed is higher than *v*_entrained_, the next pulse will occur at a later phase, decreasing the speed toward *v*_entrained_. Aside from the unimportant exception (early in double stance phase) discussed above, all the other phases with positive 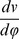 act as repellors. Accordingly, the pulses are phase-locked to the strongest attractor at the end of a double stance. The end of double stance may be regarded as the “global” attractor for phase locking associated with entrainment.

### Ankle Actuation Constant, *k*

The parameter value, *k*, was chosen to approximate the amount of ankle torque during normal human walking. The chosen value of 87.3 N·m/rad is of the same order of magnitude as the human ankle stiffness with co-contraction of ankle muscles, which is approximately 50 N·m/rad [44]. One plausible reason for the difference may be muscle activation levels. In [44] subjects were asked to maintain muscle activation at 20% of maximal activation level as indicated by electromyography (EMG) amplitude whereas in normal human walking EMG amplitudes of ankle plantar-flexion and extension muscles exceed 20% of maximal activation level: 90% for Soleus, 80% for Gastrocnemius, 40% for Posterior Tibialis, 40% for Flexor Digitorum Longus, 80% for Flexor Hallucis Longus, 40% for Peroneus Brevis, and 30% for Peroneus Longus [30]. The simplicity of the model and the ignored physiological and anatomical realism may be another source of the discrepancy. However, most importantly, comparison between the ankle actuation constant, *k* and human ankle stiffness during walking may be appropriate only when the human ankle acts as a spring that stores and releases potential energy during stance phase with a *constant equilibrium position*, which may be invalid given that lower limb muscles are actively modulated during stance.

### Limitations of This Model

To maximize simplicity, we neglected numerous aspects of locomotion. For example, multi-period gaits were not analyzed. Though multi-period asymmetric gaits are observable in human locomotion, we limited our attention to the existence and stability of a period-one gait, which represents the fundamental mode of normal gait. In the model the periodic mechanical perturbation could only accelerate gait and entrainment was only possible for perturbation periods shorter than preferred stride period *τ*_p_<2*τ*_0_. Experimentally, we observed entrainment both to faster and slower perturbations, albeit with a narrow basin of entrainment [24].

Physiological and anatomical realism was ignored by assuming a point mass body and massless legs. The massless legs significantly simplified the system dynamical equations and allowed zero torque at the hip joint. That is consistent with the experimental observation that ankle torque is the largest joint torque in normal human walking [30], [45]. The observation that the model's unperturbed average speed (1.03 m/s) was relatively slow, comparable to the speed of freely-selected slow human walking, may partly be due to the lack of actuation at hip and knee joints while the ankle actuation parameter, *k*, was determined based on normal human ankle torques during walking [33].

## Conclusion

Human walking exhibits many features associated with limit-cycle oscillators including entrainment to periodic perturbations. A finite basin of entrainment such as we observed experimentally requires a non-linear dynamical system, but there are several physiologically-plausible candidates that might be responsible. Any combination of several peripheral neuro-mechanical factors—self-sustaining oscillatory neural networks (e.g., CPGs in the spinal cord); “chaining” of reflexes based on afferent feedback; gravito-inertial dynamics of the musculo-skeletal system; and discrete, dissipative mechanical interaction with the physical environment—may exhibit limit-cycle behaviors and entrainment; and they may do so without supra-spinal control. The remarkable competence of the simple model presented here suggests that a state-determined process based on afferent feedback may be the minimal model component to describe measurable human walking behavior, in particular, asymptotically stable periodic motion and entrainment with phase locking to a narrow range of periodic perturbations.

The mechanics of the periphery accounts for a significant portion of this model's competence. Combined with minimal afferent feedback, simple peripheral mechanics can account for asymptotically stable periodic walking, entrainment to a narrow range of perturbations and phase locking. In the model, energy dissipation due to non-elastic foot-ground interaction is the key to asymptotic stability, entrainment and phase locking, suggesting that energy dissipation may be an essential element of legged animal locomotion rather than an accidental imperfection. This further suggests that intermittent collisional foot-ground interaction should be emphasized in human motor control and the design of therapeutic robots and exoskeletons to restore or assist human walking. It may not only provide essential sensory cues to coordinate locomotor patterns, but may also be a key mechanical factor that determines the stability of locomotion.
